# Aortic Root Aneurysm in an Extreme Athlete

**DOI:** 10.7759/cureus.26661

**Published:** 2022-07-08

**Authors:** Menachem Gurevitz, Andrew Weinberger, Daniel Miller

**Affiliations:** 1 Medicine, Mercy Catholic Medical Center, Philadelphia, USA; 2 Family Medicine, Touro College of Osteopathic Medicine, Brooklyn, USA; 3 Internal Medicine, Icahn School of Medicine at Mount Sinai, Queens Hospital Center, Queens, USA

**Keywords:** aortic root replacement, childhood infections, extreme exercise, aortic root dilation, thoracic aortic aneurysm

## Abstract

Aortic root aneurysms require close clinical monitoring due to the risk of dissection or rupture. While patients usually remain asymptomatic, it can occasionally be detected on physical examination as a diastolic murmur, which can be further confirmed with diagnostic imaging. The vast majority of aneurysms are found in patients with congenital or acquired conditions that compromise vascular integrity. Here, we present a case of an athletic, healthy appearing 54-year-old who was incidentally found to have a severely enlarged aneurysm requiring urgent surgical intervention.

## Introduction

According to the 2010 Guidelines for the Diagnosis and Management of Patients With Thoracic Aortic Disease [1] (a joint report by the American College of Cardiology Foundation, American Heart Association Task Force on Practice Guidelines, and several other societies), aneurysms of the aorta occur due to a wide variety of conditions, such as atherosclerosis, hypertension, syphilis, advanced age, and smoke inhalation. The guidelines further note that congenital conditions, such as bicuspid aortic valves, and connective tissue disorders, such as Marfans and Ehlers Danlos syndrome, are also known causes of aneurysms. Based on the increased incidence of aneurysms and their sequelae in these populations, primary care physicians monitor the aorta of these patients more closely than in the general population.

While it is difficult to know the true incidence of aortic aneurysms, large aneurysms, which are prone to rapid growth and rupture, are certainly very rare. In addition to our patient not being part of any of the at-risk populations described above, he engaged in excessive physical exercise, presumably putting him at an even lower risk for vascular conditions than the general population. The following case, in which appropriate workup following a physical examination led to the diagnosis and subsequent surgical treatment of a life-threatening condition, highlights the importance of a thorough and complete physical examination.

## Case presentation

A 54-year-old male presented to the Family Medicine Clinic to obtain medical clearance before undergoing hand surgery due to a recent hand injury sustained during a basketball game. Owing to what he considered to be his excessively healthy state, he had not been seen by a physician for more than 10 years. He had no surgical or psychiatric history, nor was he ever hospitalized for any reason. His past medical history was significant only for repeated childhood ear infections. He stated that he trained intensively every day and competed in several marathons and "Ironman Triathlon" tournaments. His daily exercise routine entailed spending at least 3-4 hours in the gym, which he frequented at least five times a week. He denied smoking, excessive alcohol use, illicit drug use, and sexually transmitted diseases, including syphilis.

During his physical examination, the patient was found to have a 5/6 diastolic murmur and a 3/6 systolic murmur, both heard best at the second right intercostal space. Additionally, increased pulse pressure was noted, ranging from 85 to 125 mm Hg. A thumping, bounding pulse was observed in his neck above the carotid arteries. Despite these findings, the patient denied any cardiac symptoms except for a recent decrease in exercise tolerance. Specifically, he denied chest pain, palpitations, dyspnea, orthopnea, syncope, presyncope, peripheral edema, chills, nausea, and vomiting. The patient also denied any family history of aneurysms, sudden death, or relevant genetic disorders such as Marfan or Ehlers-Danlos syndrome.

**Figure 1:**
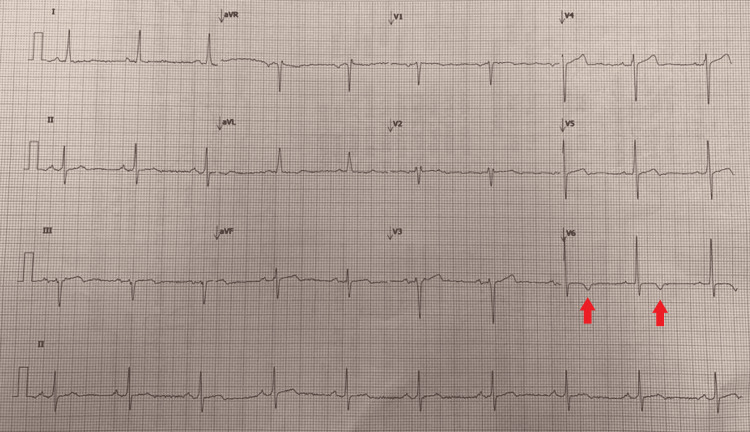
The patient's ECG depicting inverted T waves at V6 (red arrows).

On ECG, a normal sinus rhythm was observed with left axis deviation and evidence of ischemia (Figure 1). An echocardiogram showed severe aortic regurgitation, and computed tomography (CT) showed an enlarged left ventricle and dilatation of both the aortic root and ascending aorta. The specific findings observed on CT were as follows: the sinuses of Valsalva measured 57 mm by 56 mm by 54 mm, the sinotubular junction measured 66 mm, the proximal junction measured 77 mm (which was the maximal observed diameter), the mid-ascending aorta measured 70 mm, the transverse aortic arch measured 31 mm, the mid-descending aorta measured 30 mm, and at the level of the diaphragm it measured 31 mm.

Due to the nature of this severely enlarged aneurysm, the patient underwent emergent surgery (Video 1), wherein the aortic root and ascending aorta were replaced and the aortic valve was repaired. These interventions were successful, and after a short rehabilitation period, the patient was cleared to resume a limited, but progressively increasing, amount of physical exercise.

**Video 1:** The pulsating 77-mm aortic root aneurysm as seen during open surgical repair.

## Discussion

While the size of the healthy aorta varies widely in the population, according to the 2010 Guidelines for the Diagnosis and Management of Patients with Thoracic Aortic Disease guidelines mentioned earlier, an aneurysm is defined as a dilatation of the aorta greater than 50% relative to its expected diameter. The guidelines also note that the expected diameter differs among populations, with children having smaller diameters than adults, and females having smaller diameters than their male counterparts; the average diameter of the ascending aorta in an adult male is 2.86 cm. Lastly, the report notes that thoracic aortic aneurysms (TAAs) are usually asymptomatic unless it compresses surrounding structures or causes severe aortic regurgitation. Symptomatic aneurysms are usually very large and carry an extreme risk of death, and therefore there is an urgent indication of urgent surgical repair [2].

Ascending aortic aneurysms can present with aortic regurgitation, leading to heart failure, and can compress the coronary arteries leading to myocardial ischemia [3]. The incidence of diagnosed TAAs is 5.9 per 100,000 person-years [4]. The etiology of TAAs varies widely and includes bicuspid aortic valve, Loeys-Dietz syndrome, Marfan syndrome, and vascular Ehlers-Danlos syndrome, among other factors [5]. Strenuous exercise, leading to a momentary hypertensive crisis, can cause dissection or rupture of an existing aneurysm [6]. Infection has also been cited as a cause of aneurysms [7].

In a study of 230 patients with TAA, the mean aneurysmal size was 5.2 cm, the growth rate was 0.12 cm/year, and the five-year survival rate was 64% [8]. Treatment for TAAs varies and includes medical treatment, such as beta-blockers and statins for smaller aneurysms, and surgery or endovascular repair for larger aneurysms. The American Heart Association recommends screening for TAA in patients with a familial history of associated conditions, such as Marfan syndrome or a bicuspid aortic valve [3].

**Figure 2:**
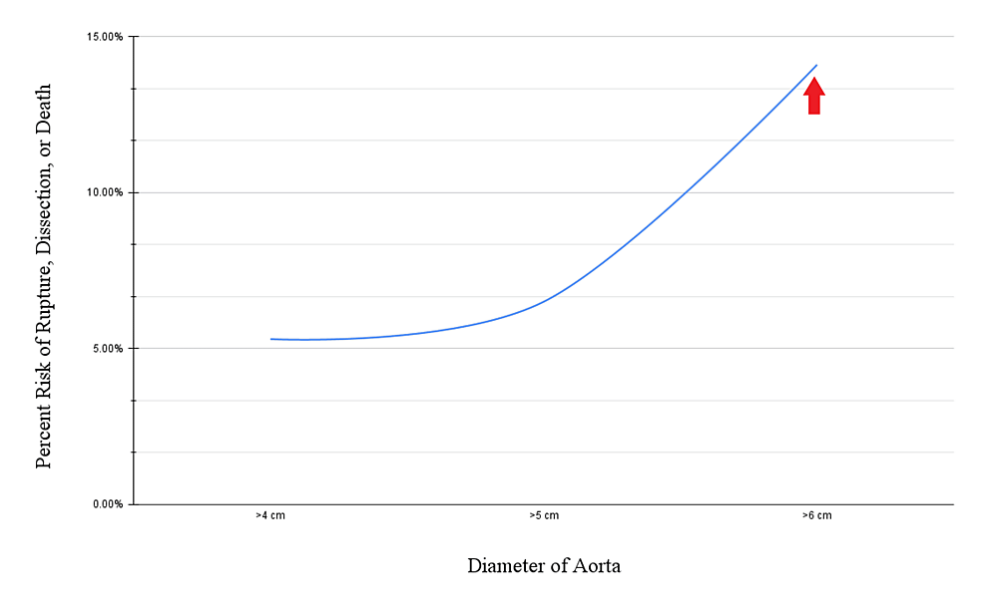
Graph depicting the combined annual risk of aortic rupture, dissection, or death as a measure of aortic diameter, with an arrow pointing to the risk corresponding to the patient in our case.

According to Agmon et al. [9], aortic aneurysms carry the risk of rupture, dissection, or death. These risks, the authors note, increase exponentially as the diameter of the aorta increases, especially beyond 6.0 cm. Additionally, according to the aforementioned study, the rate of expansion accelerates as the diameter of the aorta increases. Therefore, in addition to large aneurysms being intrinsically more dangerous, they also stand to become even larger and, therefore, even more dangerous. The risk of rupture, dissection, or death from an aortic aneurysm within one year of it being detected is illustrated in Figure 2 [10]. Note that there are little data regarding aneurysms larger than 6 cm due to their accelerated rate of expansion and resulting complications.

While we remain intrigued by the findings we presented, we propose two hypotheses that may have contributed to our patient's findings. First, the multiple infections he endured as a child may have eroded the integrity of the aortic wall, similar to how endocarditis adversely affects the aortic valves [7]. Second, excessive exercise itself, a process that generally increases cardiovascular health, may have contributed to the aneurysm. While it is well known that physical exercise can lead to cardiac remodeling and chamber wall thickening, Churchill et al. pointed to a relationship between exercise and aortic root dilation [11]. They also argue that the aorta is better thought of as a versatile organ capable of adapting to long-term stress rather than a static, unresponsive organ. Specifically, they note that "master level athletes," defined as competitive athletes who continue endurance exercise beyond their fourth decade of life, are especially likely to be found with TAAs. The finding in our patient, a master-level athlete in his sixth decade of life, is therefore consistent with what Churchill et al. reported.

## Conclusions

Aortic root aneurysms can present without any comorbid conditions. Extreme athletes may appear deceptively healthy while harboring dangerously enlarged aneurysms. As aneurysms expand, the risk of rupture or dissection increases exponentially. Therefore, it is imperative to thoroughly examine even healthy-appearing patients so that aneurysms, if present, can be diagnosed and repaired before complications arise. Possible causes of aortic root aneurysms in otherwise healthy adults include frequent childhood infections and extreme endurance exercise continued beyond the fourth decade of life.
